# A phylogeny-based sampling strategy and power calculator informs genome-wide associations study design for microbial pathogens

**DOI:** 10.1186/s13073-014-0101-7

**Published:** 2014-11-15

**Authors:** Maha R Farhat, B Jesse Shapiro, Samuel K Sheppard, Caroline Colijn, Megan Murray

**Affiliations:** Department of Pulmonary and Critical Care, Massachusetts General Hospital, Harvard Medical School, Boston, MA USA; Département de sciences biologiques, Université de Montréal, Montréal, QC Canada; Institute of Life Science, College of Medicine, Swansea University, Swansea, SA2 8PP UK; Department of Mathematics, Imperial College London, London, UK; Department of Global Health and Social Medicine, Harvard Medical School, 641 Huntington Avenue Suite 4A, Boston, MA 02115 USA; Department of Epidemiology, Harvard School of Public Health, Boston, MA USA

## Abstract

Whole genome sequencing is increasingly used to study phenotypic variation among infectious pathogens and to evaluate their relative transmissibility, virulence, and immunogenicity. To date, relatively little has been published on how and how many pathogen strains should be selected for studies associating phenotype and genotype. There are specific challenges when identifying genetic associations in bacteria which often comprise highly structured populations. Here we consider general methodological questions related to sampling and analysis focusing on clonal to moderately recombining pathogens. We propose that a matched sampling scheme constitutes an efficient study design, and provide a power calculator based on phylogenetic convergence. We demonstrate this approach by applying it to genomic datasets for two microbial pathogens: *Mycobacterium tuberculosis* and *Campylobacter* species.

**Electronic supplementary material:**

The online version of this article (doi:10.1186/s13073-014-0101-7) contains supplementary material, which is available to authorized users.

## Background

In infectious disease, host and pathogen factors interact to result in the observed severity of illness. Genetic changes within pathogen populations can result in a spectrum of virulence, drug resistance, transmission rates, and immunogenicity - all highly relevant phenotypes in the study of infectious disease. Host variables that affect susceptibility to infection, such as age, immunodeficiency, and nutritional status are more easily measured and have been studied for some time, whereas the study of pathogen specific determinants of disease risk is more recent. One of the first to use the term molecular epidemiology and apply it to infectious disease agents was E. Kilbourne. In his 1973 paper ‘Molecular epidemiology of influenza’, he discussed antigenic variation as a cause of the influenza pandemics of the 20th century [1]. The ability to type molecular traits of pathogens, such as surface proteins or highly variable DNA segments, allowed the characterization of sufficient strain-to-strain variation to determine when transmission of disease occurred [2] as well as surveillance of the frequencies of different strain types over time [3]. As sequencing became sufficiently high throughput to allow for whole genome analysis, the typing resolution immediately reached the limit for heritable strain differences and has accordingly gained momentum in the study of infectious disease [4-7].

Molecular epidemiologic tools have not only enabled disease surveillance and the study of transmission chains, but also have facilitated the study of pathogen biology, by allowing researchers to compare the transmissibility, immunogenicity, or other phenotypes that vary among strain types or lineages and correlate these differences with specific changes in the genome [8,9]. Large numbers of pathogen samples are often gathered for clinical diagnostic purposes. For pathogens of high outbreak potential, samples may be collected for surveillance purposes. The short evolutionary times corresponding to outbreaks often mean that samples of transmitted pathogens are clonal. The availability of samples from diagnostic and outbreak setting, and the DNA sequences generated from them, means that investigators are faced with questions about which and how many pathogen isolates to sequence and which analytical techniques to use to maximize efficiency and power. These questions are especially relevant for studies of whole-genome sequences (WGS) that will generate thousands of potentially relevant mutations, the great majority of which will be noise, that is, neutral mutations not related to the phenotype of interest.

The methods underlying human genome-wide association studies (GWAS) and whole exome sequencing have advanced significantly in the past 10 years, and are now more rigorous and standardized across studies of different human traits and diseases [10,11]. These advancements have included recommendations on study design including subject selection strategies and sample size to uncover elements of varying frequency and effect sizes. These methods are most well developed for single nucleotide polymorphism (SNP) changes in typing data (as opposed to whole genome sequences) and make implicit assumptions about the human genomic structure, diploidy, and recombination rates [12-14]. The situation is different in bacteria where recombination and genetic mutation rates vary among species, from highly clonal organisms like *Mycobacterium tuberculosis* (MTB), to the rapidly recombining/sexual *Streptococcus pneumoniae*. In contrast to disease states in humans, pathogen phenotypes of interest are often those that provide a selective advantage for the organism. Several different methods are in current use for the study of genome wide variation of pathogens that, in contrast to human genetic association studies, can frequently leverage information about positive selection. Despite this, the field has not yet defined accepted methodologies and standards for statistical testing of variants on a whole genome scale. In this paper we review the literature on genotype-phenotype studies and analytical techniques focusing on MTB as an example. We propose a matched genome sampling and analysis strategy to optimize power for pathogens that are clonal to moderately sexual. We provide an associated power and sample size calculator and demonstrate and validate the method using two genomic datasets: one from MTB and one from *Campylobacter* species.

## Methods

The methods outlined below were used for the application of the sampling strategy.

Strain isolation, culture, sequencing, and variant calling are detailed in the original publications [15,16].

### Phylogeny construction

MTB: The phylogeny was constructed based on the whole genome multiple alignment. As MTB populations are considered to be predominantly clonal, most of the genome is thought to support a single consensus phylogeny that is not impacted significantly by recombination [17]. A superset of SNPs relative to reference strain H37Rv [18] was created across the clinical isolates from the variant caller SNP reports. SNPs occurring in repetitive elements including transposases, PE/PPE/PGRS genes, and phiRV1 members (273 genes, 10% of genome) (genes listed in reference [19]) were excluded to avoid any concern about inaccuracies in the read alignment in those portions of the genome. Furthermore, SNPs in an additional 39 genes previously associated with drug resistance [20] were also removed to exclude the possibility that homoplasy of drug resistance mutations would significantly alter the phylogeny. After applying these filters the remaining SNPs were concatenated and used to construct a parsimony phylogenetic tree using PHYLIP dnapars algorithm v3.68 [21] with KZN-DS [22] strain as an outgroup root. We constructed a phylogeny by two methods. First, using Bayesian Markov chain Monte Carlo (MCMC) methods as implemented in the package MrBayes v3.2 [23] using the GTR model and a maximum likelihood tree using PhyML v3.0 [24]. Second, using the GTR model with eight categories for the gamma model and the results were consistent with the PHYLIP Phylogeny.

### Campylobacter

Using multi-local sequence typing data, a phylogeny was estimated using ClonalFrame [25], a model-based approach to determining microevolution in bacteria. This program differentiates mutation and recombination event on each branch of the tree based on the density of polymorphisms. ClonalFrame was run with 50,000 burn in iterations and 50,000 sampling iterations. The consensus tree represents combined data from three independent runs with 75% consensus required for inference of relatedness. Recombination events were defined as sequences with a length of >50 bp with a probability of recombination > =75% over the length, reaching 95% in at least one site.

### Analysis

The number of mutations, insertions, or deletions (of any size) differing between each strain pair was summed across each locus for the eight strain pairs for each of the two datasets belonging to MTB or *Campylobacter*. The upper 95% confidence interval for the average number of mutations/locus across the eight pairs was used as a mean of the null Poisson distribution. All genes with larger counts than expected under this null distribution were considered to be significantly association with the resistance phenotype.

## Results and Discussion

### Literature search

We first defined five cornerstones of a systematically designed microbial genotype-phenotype association study: (1) a well-defined phenotype of interest, that can be measured/classified with negligible error; (2) some understanding of the effect size for that phenotype, for example is it influenced by many genetic variants each with small or incremental effect, or are there fewer variants with a large effect?; (3) estimates of the number of whole genomes needed to achieve nominal power; (4) a sampling strategy that may include the sequencing of pathogens serially sampled over time from the same patient, the study of strains matched by some predefined characteristic, a ‘random’ subsample, or an ‘exhaustive’ complete sample; and (5) a defined statistical analysis strategy that maximizes power and minimizes the rate of false positives.

**Figure 1:**
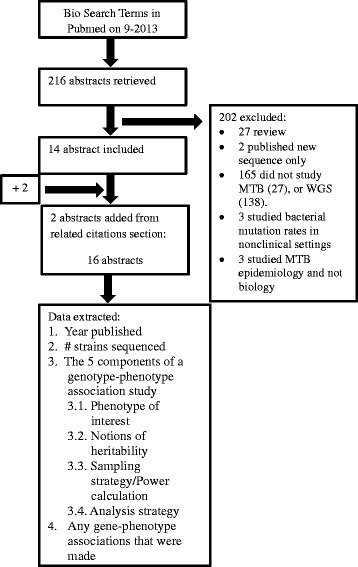
**Flow chart detailing literature search.**

We performed a systematic search of the literature to determine which sampling and analytical strategies (the five components above) have been applied to the study of MTB biology using whole genome sequences. We sought articles studying one of the following aspects of MTB biology: immunogenicity, pathogenicity, virulence, transmissibility, drug resistance, or fitness using whole genome sequences. Search terms, inclusion and exclusion criteria are detailed in Table 1. We searched PubMed on 1 September 2013 and identified 216 abstracts, and included 16 studies (Figure 1, Table 2).

**Table 1:** **PubMed Search terms and inclusion and exclusion criteria**

**Search purpose**	**Search terms**	**Inclusion criteria**	**Exclusion criteria**
Identify studies of Pathogen Biology using whole genome sequencing and analysis	‘genome sequencing’ AND ‘tuberculosis’ AND (‘drug resistance’ OR ‘virulence’ OR ‘immunogenicity’ OR ‘transmissibility’ OR ‘fitness’)	All abstracts describing the use of WGS data to identify genes related to pathogen immunogenicity, virulence, transmissibility, drug resistance, or fitness	(1) Review articles
			(2) Studies that published new sequence data only
			(3) Studies that did not study MTB bacteria and its biology
	In all PubMed fields		
			(4) Studies that only assess mutation rates in non-clinical settings

**Table 2:** **Literature search results**

**Author**	**Reference**	**Year**	**Strains sequenced (n)**	**Stated study purpose** ^**a**^	**Clinical strains?**	**Time series?**	**Method**	**Report specific genotypic association?**
Zhang *et al.*	[26]	2013	161	Identify drug resistance genes	Yes	No	Phylogenetics and comparison of rates with poisson distribution	Yes; list of genes provided
Farhat *et al.*	[15]	2013	124	Identify drug resistance genes	Yes	No	Phylogenetics and convergence analysis	Yes; list of genes provided
Lin *et al.*	[27]	2013	2	Identify drug resistance genes	Yes	No	Comparison with reference mycobacterial strains	No
Wu *et al*.	[28]	2013	4	Identify Beijing associated pathways	Yes	No	COG enrichment of genes with snps	General pathways rather than individual genes
Das *et al.*	[29]	2013	5	Identify genes related to extrapulmonary TB	Yes	No	COG enrichment of genes with snps	General pathways rather than individual genes
Ilina *et al.*	[30]	2013	4	Identify drug resistance genes	Yes	No	Comparison with reference mycobacterial strains	No
Abrahams *et al.*	[31]	2013	-	Identify resistance targets(s) for novel imidazole	No	Yes: spontaneous mutants resistant to drug and their sensitive ancestor	Identification of all mutations	Yes *qcrb*
Supply *et al.*	[32]	2013	5	Identify genes associated with smooth TB phenotype	Yes	No	Comparison with reference mycobacterial strains	General pathways rather than individual genes
Hartkoorn *et al.*	[33]	2012	-	Identify resistance targets(s) for pyridomycin	No	Yes: spontaneous mutants resistant to drug and their sensitive ancestor	Identification of all mutations	Yes acyl-carrier-protein *inha*
G. Sun *et al.*	[34]	2012	7	Identify drug resistance genes	Yes	Yes: serial samples from same patient	Identification of all mutations	No; but list of potential candidates with new fixed mutations provided
Grzegorzewicz *et al.*	[35]	2012	-	Identify resistance targets(s) for novel compound Adamantyl Urea	Yes	Yes: serial samples from the same patient	Identification of all mutations	Yes *mmpl3*
Casali *et al.*	[36]	2012	59	Identify drug resistance genes	Yes	No	Phylogenetic tree and parallel evolution and convergence	Yes *rpoc*
Tahlan *et al.*	[37]	2012	-	Identify resistance targets(s) for novel compound SQ109	No	Yes: spontaneous mutants resistant to drug and their sensitive ancestor	Identification of all mutations	Yes *mmpl3*
La Rosa *et al.*	[38]	2012	-	Identify resistance target(s) for 1,5-diarylpyrrole derivative BM212	No	Yes: spontaneous mutants resistant to drug and their sensitive ancestor	Identification of all mutations	Yes *mmpl3*
Comas *et al.*	[39]	2011	10	Identify drug resistance genes	Yes	Yes: serial samples from the same patient	Identification of all mutations in rpoc. Assessment of convergence across different strain pairs	Yes confirmed rpoc
Manjunatha *et al.*	[40]	2006	-	Identify resistance targets(s) for PA-824	No	Yes: spontaneous mutants resistant to drug and their sensitive ancestor	Identification of all mutations	Yes Rv3547

^a^The term ‘phenotype related genes’ is used loosely here to describe genes that are associated with but not necessarily causative of the phenotype.

#### Phenotype

Most of the studies (13/16) focused on the MTB resistance phenotype to a wide range of drugs. Three other studies examined other strains including: (1) strains causing extrapulmonary tuberculosis; (2) strains with a smooth phenotype; and (3) strains typed as Beijing using spoligotyping.

Effect sizes and *a priori* power calculations were not explicitly discussed in any of these studies.

#### Sampling

Half of the 16 studies sampled strains in time-course, either in laboratory-evolved strains (five studies), or in serial samples from the same patient (three studies). In all cases, strains were initially drug sensitive but later acquired a drug resistance phenotype. In the other eight studies, clinical MTB samples were obtained from different TB patients, and generally involved the study of more distantly-related strains than in the time-course studies. In general strains were sampled more or less randomly to include strains with and without the phenotype. Seven of the non-time-course studies were published within the last year.

#### Analysis

In the time-course studies, few mutations occurred and it was generally tractable to identify all novel mutations and infer their role in resistance. In the other studies, only two of eight were able to make specific genomic associations supported by formal assessments of statistical significance; both these studies sequenced a relatively large number of genomes (>100), and used phylogenetic ancestral reconstruction in their analysis of mutations relevant to the phenotype [15,26]. Two studies [15,36] used phylogenetic convergence (described below) to select candidates for association with the drug resistance phenotype. In the other six studies, the phenotype-genotype associations were of a more descriptive, less formal nature.

Across all studies, a common theme was the use of tests for positive selection and phylogenetics to differentiate between genetic variation related to strain ancestry and those relevant to the phenotype [15,36]. There are also examples from non-TB pathogens [16,41]. In the phylogenetic convergence test mentioned above, a relatedness tree, constructed using the whole genome data is used to identify genes that accumulate frequent mutations synchronous with the acquisition of the phenotype of interest. Phylogenetic convergence has several advantages well-suited to the study of microorganisms. Most notably, by focusing only on the genetic changes that coincide with the independent appearances of the phenotype, it ignores false-positive associations due to clonal population structure, namely the genetic relatedness of the strains [15,16,36,41,42]. It can therefore be applied to both clonal and sexual/recombining pathogens as long as recombination is taken into account in the phylogenetic tree construction [43]. For highly recombining pathogens, the tools of human GWAS might be appropriate, with some modifications [44,45].

### Sampling and analysis strategy

The literature review highlights the success of time-course WGS, either within patients or *in vitro*, to identify the genetic bases of clinically-important phenotypes. However time-course samples are often difficult to obtain, particularly in clinical settings, and may not always be generalizable to the larger population of pathogens [46]. In contrast to time-courses, ‘cross-sectional’ samples of strains routinely collected for patient diagnosis or public health surveillance are both easier to obtain and may provide a more comprehensive, global picture of a pathogen’s adaptive landscape.

A major challenge posed by studying diverse clinical strains is that the sampled population of pathogens may contain population structure related to the shared ancestry of the strains. Populations are considered structured when they include subpopulations among which the frequency of genotypes differs systematically. Population structure, a form of non-independence of observations, can be seen when pathogen strains are isolated from disease outbreaks or direct transmission chains, or clusters, and compared with non-clustered strains; The study of pathogen subpopulations when they also preferentially share the phenotype of interest, can lead investigators to wrongly associate the subpopulation genotype, shared by virtue of ancestry alone, with the phenotype of interest. This type of confounding bias is a well-recognized problem in human GWAS [11,47-49].

Whereas different methods such as Principle Components analysis, mixed effects models and phylogenetic convergence can be used to correct for population structure [11,47-51], adopting a careful sampling strategy can minimize the impact of - or even capitalize on - population structure. Drawing parallels from case-control study design in epidemiology and human GWAS [47] we propose that sampling ‘matched’ pairs of closely-related strains with different phenotypes can not only control for population structure but can also deliver higher power relative to sampling randomly from strain collections. The matching procedure we propose addresses population structure and improves power by ignoring the shared variants within a subpopulation and focusing only on the recently evolved differences, thus reducing the number of variables tested and improving power. The sequence data generated using matched sampling can be analyzed using a simplified form of phylogenetic convergence by: (1) identifying the recently evolved mutations by pairwise alignment of a sequence from a strain with the phenotype of interest with a closely-related strain lacking the phenotype; (2) counting the number of mutations across several such pairs; and (3) comparing these counts either to a null distribution generated using a non-parametric permutation test [15], or simply to a Poisson or Binomial distribution, as we will discuss and demonstrate in the next sections.

**Figure 2:**
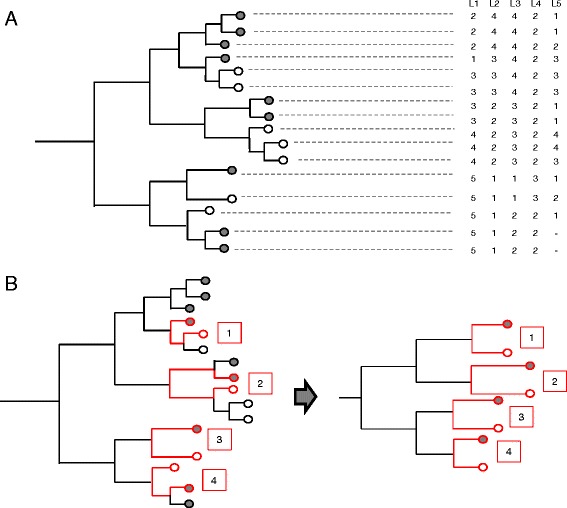
**Demonstration of the selection strategy. (A)** Example initial MIRU-VNTR phylogeny constructed for selection of strains for sequencing and analysis. Grey circles represent strains with the phenotype of interest (ph+ strains), the white circles represent strains without the phenotype of interest (ph- strains). The Table with columns L1-5 represent the variable number of tandem repeat at each locus L. **(B)** Example of selection methodology: For each ph+ strain (grey circle) a neighboring ph- strain is selected such that the distance between the two strains in the phylogeny is minimized. Each control or study strain is only sampled once. The resultant tree of selected strains will consist of matched study and control strains.

Assuming a binary phenotype of interest that has been clearly defined, we propose to match strains using data from traditional strain typing such as pulsed-field gel electrophoresis and multi-locus sequence typing that is often already available for the banked strains, especially under surveillance for public health purposes. Using this lower resolution typing data, a phylogenetic tree can be constructed, accounting for recombination as needed using methods such as ClonalFrame [16,25]. Figure 2A displays a hypothetical tree topology obtained for a sample of 16 MTB clinical strains constructed using their MIRU-VNTR pattern [52]. Figure 2B demonstrates the matched sampling strategy. For each phenotype positive (ph+) strain, a neighboring phenotype negative (ph-) strain is selected such that the phylogenetic distance between the pair of strains is minimized. Only one ph- and one ph+ strain is sampled per clade. If more than one strain is equidistant, then one is selected at random. The larger phylogenetic tree is thus reduced to a set of matched ph+ and ph- pairs.

### Power calculations to optimize genotype-phenotype association studies

To design a genotype-phenotype association study, knowledge about the optimal number of pathogen genomes to sample is necessary. Here we define the sample size *n* as the number of matched genome pairs necessary to achieve a nominal power of >80% for detecting a true association, accepting a false positive association rate of no higher than 0.05. Our goal is to identify genomic variants, for example mutations or recombination events that confer a fitness advantage when the phenotype of interest such as antibiotic resistance, virulence, evolves under selective pressure. These positively selected variants are expected to be more prevalent in strains with the phenotype of interest (ph+). Below, we will describe two methods to identify genomic variants associated with this phenotype of interest. The first, ‘site-level’ method, uses individual nucleotide sites as the basic level of genetic variation. However, this method can also be applied to other levels of variation, including the presence of absence of genes, or clusters of mutations that are transferred together by recombination and can thus be considered as a unit. This method is therefore applicable to clonal pathogens that evolve almost entirely by point mutation, as well as to moderately recombining pathogens, in which recombinant parts of the genome can be identified computationally [53-55] and considered as a single ‘site’. In the second, ‘locus-level’ method, we model a scenario in which different mutations within the same gene or locus can have a similar phenotypic effect, for example the loss of function by introducing stop codons at different points in the gene, providing additional evidence for the importance of that gene for a particular phenotype.

In the site-level method, for an organism with genome of length *k* and an average distance (or number of variants) *s* between each pair of strains, we can define a null hypothesis for the distribution of the number of variants *l*_*j*_ at a particular neutral site (*j*) in the genome (in the ph+ relative to the ph- strains) across the *n* pairs. In particular, if the site *j* is not under selection, then *s/k* should be a reasonable estimate of the rate of neutral variation, and under the null hypothesis, *l*_*j*_ is a Binomial random variable corresponding to *n* trials with a success probability *p*_*Null*_ *= s/k.* Under the alternative hypothesis that site *j* is under positive selection, *l*_*j*_ is a binomial random variable with *n* trials and success probability *f*_*site*_ which is greater than *s/k. f*_*site*_ is related to the phenotypic effect size of the variant, as a higher frequency of a variant will result from stronger positive selection, that is, higher fitness of the variant in ph+ relative to ph- strains [56]. An extreme example would be a selective sweep that results in all members of the ph+ population carrying the same variant in which case *f*_*site*_ would be 1. In a previous genotype-phenotype association study of drug resistance in MTB [15], the lowest frequency of a single nucleotide (‘site level’) variant with a known fitness advantage was estimated at 4% (*f* = 0.04) (*rpoB* codon 455 in rifampicin (RIF) resistant strains), whereas the highest was estimated at 52% (*f* = 0.52) (*rpoB* codon 450).

**Figure 3:**
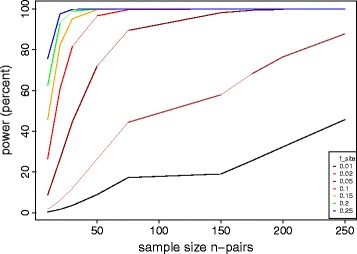
**Power of the matched convergence test for identify nucleotide sites associated with a phenotype of interest.** The average genetic distance between matched strains was set to an intermediate level of s = 100 mutations. Colors represent increasing values of site effect size *f*_*site*_.

**Figure 4:**
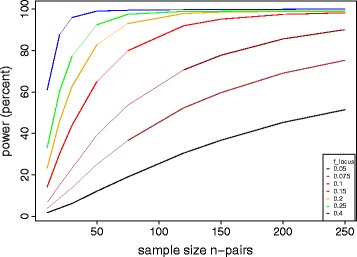
**Power of the matched convergence test to identify loci associated with a phenotype of interest.** The average distance between matched strains was set at s = 100 mutations. Colors represent increasing values of locus effect size *f*_*locus*_.

**Figure 5:**
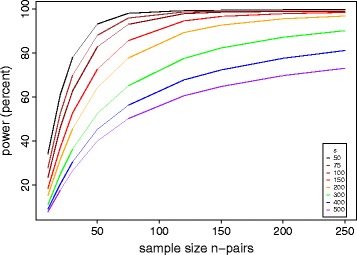
**Power of the matched convergence test at the locus level as a function of genetic distance (** ***s*** **) between matched strains pairs.** Smaller *s* indicates closer genetic relatedness between strain pairs.

As observed for *rpoB*, more than one nucleotide site in a locus can carry a fitness conferring variant; we can thus formulate a locus-level test by defining a null distribution for the sum of the variant counts in a locus, *l*_*i_locus*_. If locus *i* of length *g*_*i*_ is not under selection, with the same parameters *s* and *k* defined above, then the distribution of *l*_*i_locus*_ can be approximated by a Poisson distribution with a rate = *n s g*_*i*_*/k*. Under the alternative hypothesis, this locus is under selection and the expected number of mutations is *n f*_*locus*_, which is larger than *n s g*_*i*_*/k*. Similar to *f*_*site*_, *f*_*locus*_ is related to the collective fitness advantage conferred by its variants. For example, in the study cited above, *f*_locus_ was estimated to be 0.30 to 1.5/locus/ph+ strain for the *thyA* locus for MTB p-aminosalicylic resistance, and *rpoB* locus for RIF resistance, respectively [15]. The test will have a different power for different values of *f*_*site/locus*._ Because this analysis involves testing all the sites and loci with observed variation, a correction for multiple testing is needed. We use the Bonferroni correction, assuming that the upper limit for the number of variable sites across the sample is *n s*, and the number of variable loci to be *1- e*^*-n g*^_*i*_^*s/k*^ (from the Poisson distribution). In Figures 3, 4, and 5, we provide power calculation results as a function of *n*, *s* and *f* using the 4.41 Mbp MTB genome as an example. Here we calculated the expected power by integrating across the distribution of locus lengths *g*_*i*_ for the MTB reference genome H37Rv. Based on previous data from fingerprint-matched MTB, our power calculations explored a range of between-strain genetic distances (*s*) from 50 to 300 mutations [4].

In the case of MTB, we found that high power (>80%) could be achieved by sequencing 50 to 100 strain pairs (matched at a distance of *s* = 100 variants) to detect a ‘rare’ drug resistance variant in >5% of the ph+ strains (*f*_*site*_ >0.05; Figure 3) or a locus with a low mutation rate of 0.25/locus/ph+ strain (*f*_*locus*_ >0.25; Figure 4). The advantage of performing a locus-level analysis is that we expect *f*_*locus*_ > *f*_*site*_ because *f*_*locus*_ is proportional to the sum of *f*_*site*_ over all sites under selection in the locus. The number of tests performed in a locus-level analysis is several orders of magnitude lower than with a site-level analysis because a bacterial genome contains on the order of 10^6^ sites, but only 10^3^ genes (loci). We performed similar calculations for *Campylobacter* (*k* = 1.64 Mbp), assuming a higher matching distance *s* = 300 that is expected for multi-locus sequence typing (MLST) of this pathogen [16]. With 50 to 100 strain pairs of Campylobacter the lowest *f*_*locus*_ that can be detected with >80% power is 0.60 (Additional file 1: Figure S1), higher than for MTB (Figure 4).

We next explored how power depends on the genetic distance between sampled genomes. Figure 5 demonstrates that considerable power gains can be achieved by sampling strain pairs that are close genetic relatives (low *s*). This is because, for a given value of *f*_*site*_ or *f*_*locus*_, raising *s* decreases the ratio of selected to neutral variants, thereby decreasing the signal to noise ratio.

The power calculator is provided with this manuscript as an R function (Additional file 2), and allows the user to tune all the parameters described to provide power estimates for different effect sizes, different pathogen genome sizes, and different levels of genetic relatedness.

### Application to genomic data from MTB and *Campylobacter* species

**Figure 6:**
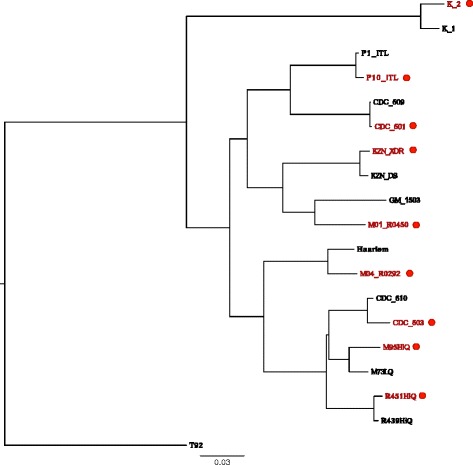
**Phylogeny of MTB strains chosen for genotype-phenotype analysis.** Dots indicate the presence of the drug resistant phenotype. The tree demonstrates the matching of strains with and without the drug resistance phenotype.

**Figure 7:**
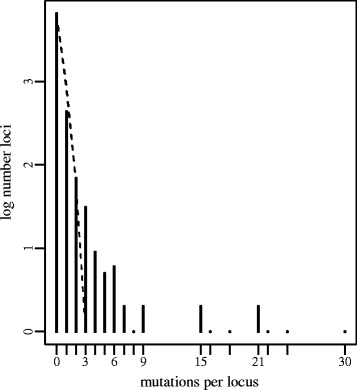
**Distribution of SNPs/locus across the eight pairs of MTB genomes.** Observed counts are represented by black bars. The dashed line represents the upper 95% confidence bounds on a Poisson distribution with the observed number of mutations.

We applied the sampling strategy described in Figure 2 to a set of 123 clinically isolated unmatched *MTB* genomes previously analyzed using phylogenetic convergence [15] (Additional files 3 and 4). Repetitive, transposon, and phage-related regions were removed as putatively recombinant or as error-prone regions of the alignment. Of the 123 strains, 47 were resistant to one or more drugs (ph+) and the rest were sensitive (ph-). As different fingerprinting methods were used for the different strains in this study and for demonstration purposes we used the phylogeny constructed using whole genome single nucleotide polymorphisms to match strains. We chose eight pairs of strains using this selection strategy (Figure 6). We then counted the recent mutational changes (single nucleotide polymorphisms; SNPs) between each pair of strains. The average distance (*s*) between pairs was 109 SNPs and was in the range of 12 to 254 SNPs. We calculated the number of changes per gene across the eight pairs and compared this number to a Poisson distribution of mutations randomly distributed across branches as the null distribution. We then identified the tail of the distribution, containing genes with a high number of changes highly associated with drug resistance (Figure 7). Overall, 12 genes and non-coding regions were found to be associated with drug resistance using only 16 out of 123 strains (13%) used in the original analysis. The analysis identified *katG*, *embB*, *rpoB* (well known drug resistance determinants) as well as top new candidates from the previous full analysis of all 123 genomes: *ponA1*, *ppsA*, *murD*, and *rbsk*. This selection strategy and analysis recovered 67% of the candidates identified with the full analysis, but used only 13% of the data, demonstrating the superior power of the matched convergence analysis to the general unmatched test.

**Figure 8:**
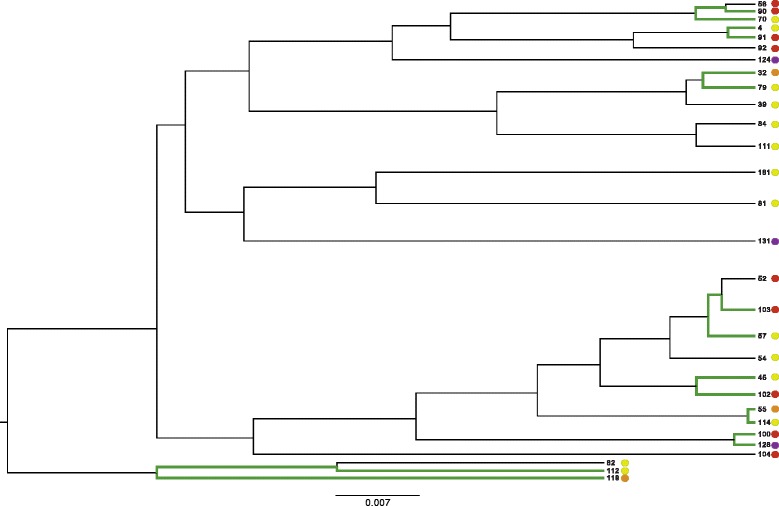
**Phylogeny of Campylobacter strains.** Branches highlighted in green lead up to the strain pairs chosen for genotype-phenotype association. Colored circles denote host specificity: red = cattle, green = chicken, purple = wild bird/non-host, orange = human.

**Figure 9:**
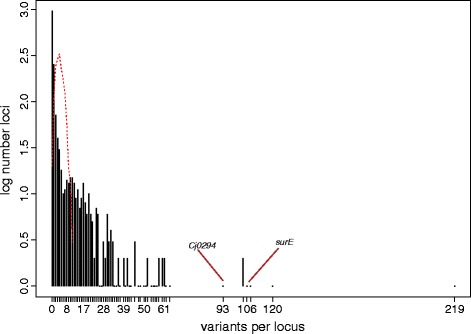
**Distribution of variants/locus across the eight pairs of** ***Campylobacter*** **genomes.** Observed counts are represented by black bars. The dashed red line represents the upper 95% confidence bounds on a Poisson distribution with the observed number of variants. Variant counts per locus for *surE* and *Cj0294* are highlighted.

Second, we applied the same method to a set of 192 *Campylobacter coli* and *jejuni* isolates used by Sheppard *et al.* in an association study to identify the factors responsible for adaptation to cattle and chickens [16] (Additional files 5 and 6). Sheppard *et al.* associated the presence or absence of unique 30 bp ‘words’ with the host specificity phenotype and controlled for population structure by comparing the real word counts with word counts generated along the tree through Monte Carlo simulations. We applied our method to a subset of 29 strains enriched in the phenotype of host switching that Sheppard *et al.* had used in their initial analysis. After correcting for recombination and constructing the phylogeny using ClonalFrame, we phylogenetically matched 8 pairs of strains that had undergone host switching (Figure 8). Five switches were estimated from cattle to bird or human, and three were from bird to human hosts. We counted the pairwise differences across the eight pairs, grouping insertions/deletions and mutations by gene and compared the distribution to the expected Poisson distribution (Figure 9). We associated two consecutive genes: *surE* and *Cj0294*, both of which were present in cattle-associated strains but absent in chicken-associated strains. These genes mapped to a vitamin B5 biosynthesis region, which Sheppard *et al.* had previously found to affect *Campylobacter* growth in the presence or absence of vitamin B5 [16]. In addition, our approach associated 105 additional genes (Additional file 7: Table S1). Thus, using the convergence method and focusing on genes rather than 30 bp words, we were able to detect the experimentally-validated vitamin B5 region of the *Campylobacter* genome, among other potential genes involved in host switching that had been observed by Sheppard *et al.* using a much smaller dataset.

Our power calculations rely on a well-defined phenotype that can be measured without error. The phenotype is also assumed to be binary, or at least divisible into two binary states; therefore, the calculations cannot be easily extended to quantitative traits. Knowledge about the expected effect size for different phenotypes is also important for these calculations and prospective study design. Among the studies reviewed, we found the effect size to be infrequently reported for MTB. Here we provide empirical effect sizes estimated from a previous MTB drug resistance study [15] as a reference point for future studies.

Our approach also assumes that a certain amount of previously collected antigen or genotyping data is available to allow for building a phylogeny and selecting pairs of strains to sequence. When sequence data are already available, this method can still be used to select strains for paired analysis, providing a simple control for population structure and a more simplified analysis strategy. If no typing data are available, alternatives may still exist - for example, using epidemiological data that link strains within a particular outbreak. In each of these scenarios, perfect matching to form pairs of monophyletic strains may not always be possible, but given the relationship of the matching distance to power demonstrated above, we argue for matching as many strains as possible and as closely as possible. The analysis of the total dataset of all monophyletic and paraphyletic pairs can be performed via ancestral reconstruction and a more general phylogenetic convergence method (‘phyC’ [15]) rather than the simplified pairwise analysis described here.

Our power calculations, like all models, make necessary simplifications and assumptions. For example, we assume that neutral variants are distributed randomly across the whole genome. This may not necessarily be the case as some pathogen genes may contain mutation or recombination hot spots. Some adjustment for such a scenario could be made by using a higher average rate of variation than the one expected, that is, testing power under a pairwise distance *s*^*†*^ amplified by a factor *m > 1* where *s*^*†*^ *= m s*_*expected*_ for a range of *m*. The framework and power calculations presented here represent a step toward more systematic and prospective genotype-phenotype study design for microbial pathogens, and can provide the basis for more refined power calculations (for example, accounting for continuous rather than binary phenotypes, or for analysis of un-matched strains).

## Conclusions

The improved ability to study the evolution of clinical strains will be an important advance for the study of pathogens as they spread. Thus far, most of our understanding of infectious disease has focused on the epidemiological study of host risk factors, or on the *in vitro* study of the pathogen. The rich information contained in whole genomes of clinical pathogens - isolated as they adapt to their host and cause disease - provides a new and complementary perspective on pathogen biology. Here we have shown how clonal to moderately sexual strain collections, originally assembled for epidemiological purposes, using appropriate sub-sampling schemes, can empower genome-level association studies and reveal genotype-phenotype associations, increasing our understanding of pathogen biology and adaptation.

## Additional files

Additional file 1: Figure S1. Power of matched convergence test to identify phenotype associated loci. The average distance between matched strains was set at s = 300 variants. Colors represent increasing values of locus effect size *f*_*locus*_. Additional file 2:
**Power calculator as R function.**
Additional file 3:
***Mycobacterium tuberculosis*** **strains multiple sequence alignment file.**
Additional file 4:
***Mycobacterium tuberculosis*** **strains drug resistance profile.**
Additional file 5:
***Campylobacter*** **strain multiple sequence alignment file.**
Additional file 6:
***Campylobacter*** **strain host specificity phenotype.**
Additional file 7: Table S1.
*Campylobacter* genes associated with host switching using the matched selection strategy and the proposed analysis.

## Abbreviations

GTR: Generalized Time Reversible substitution model
GWAS: Genome Wide Association Study
MIRU-VNTR: Mycobacterial interspersed repetitive units-variable number tandem repeats
MLST: Multi-locus sequence typing
MTB: Mycobacterium tuberculosis
SNPs: Single nucleotide changes
TB: Tuberculosis
WGS: Whole-genome sequencing or sequences
