# Supplementary figures and images for: A phylogeny-based sampling strategy and power calculator informs genome-wide associations study design for microbial pathogens
Source: Genome Med. 2014 Nov 15;6(11):101. doi: 10.1186/s13073-014-0101-7 (PMC4256898; doi:10.1186/s13073-014-0101-7)

**Locus Level Power by effect size (f\_locus)**  
**s=300**

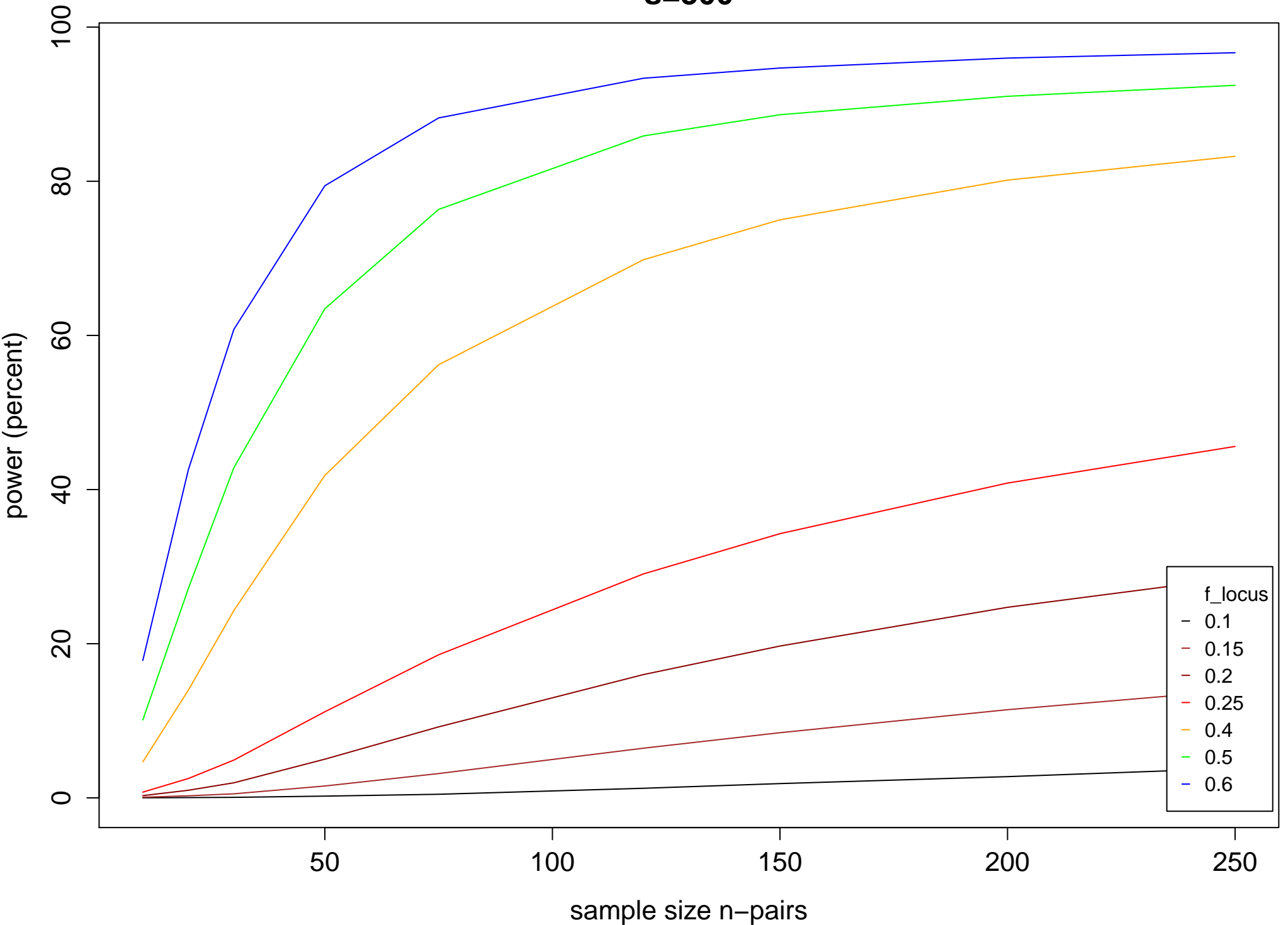

Supplement: Additional file 1: Figure S1. — Power of matched convergence test to identify phenotype associated loci. The average distance between matched strains was set at s = 300 variants. Colors represent increasing values of locus effect size f locus. [file 13073_2014_101_MOESM1_ESM.pdf]
